# Effects of Dietary Supplementation of Solubles from Shredded, Steam-Exploded Pine Particles on the Performance and Cecum Microbiota of Acute Heat-Stressed Broilers

**DOI:** 10.3390/microorganisms10091795

**Published:** 2022-09-06

**Authors:** Akshat Goel, Chris-Major Ncho, Chae-Mi Jeong, Vaishali Gupta, Ji-Young Jung, Si-Young Ha, Jae-Kyung Yang, Yang-Ho Choi

**Affiliations:** 1 Department of Animal Science, Gyeongsang National University, Jinju 52828, Korea; 2 Institute of Agriculture and Life Sciences, Gyeongsang National University, Jinju 52828, Korea; 3 Division of Applied Life Sciences (BK21 Plus Program), Gyeongsang National University, Jinju 52828, Korea; 4 Department of Environmental Materials Science, Gyeongsang National University, Jinju 52828, Korea

**Keywords:** acute heat stress, broiler chickens, cecum metagenome, performance, solubles from shredded, steam-exploded pine particles (SSPPs)

## Abstract

Heat stress (HS) negatively influences livestock productivity, but it can be, at least in part, mitigated by nutritional interventions. One such intervention is to use byproducts from various sources that are likely to be included in the consumer chain. Thus, the present study investigated the effects of dietary supplementation of solubles from shredded, steam-exploded pine particles (SSPPs) on the performance and cecum microbiota in broilers subjected to acute HS. One-week-old Ross 308 broilers (n = 108) were fed 0%, 0.1%, or 0.4% SSPP in their diets. On the 37th day, forty birds were allocated to one of four groups; namely, a group fed a control diet without SSPPs at thermoneutral temperature (NT) (0% NT) and acute heat-stressed birds with 0% (0% HS), 0.1% (0.1% HS), and 0.4% (0.4% HS) SSPP-supplemented diets. The NT was maintained at 21.0 °C, while the HS room was increased to 31 °C. The final BW, percent difference in body weight (PDBW), and feed intake (FI) were lower in HS birds, but PDBW was reversely associated with dietary SSPP. Similarly, HS birds had a higher rectal temperature (RT) and ΔT in comparison to birds kept at NT. The FI of SSPP-supplemented birds was not significant, indicating lower HS effects. Plasma triglyceride was decreased in HS birds but not affected in 0.1% HS birds in comparison to 0% NT birds. OTUs and Chao1 were increased by 0.1% HS compared to 0% NT. Unweighted Unifrac distances for 0.1% HS were different from 0% NT and 0.4% HS. The favorable bacterial phylum (*Tenericutes*) and genera (*Faecalibacterium* and *Anaerofustis*) were increased, while the pathogenic genus (*Enterococcus*) was decreased, in SSPP-supplemented birds. In sum, production performances are negatively affected under acute HS. Dietary supplementation of SSPPs is beneficial for improving community richness indices and unweighted Unifrac distances, and it enhanced the advantageous bacterial phyla and reduced virulent genera and triglyceride hydrolysis in acute HS broilers. Our results indicate that dietary SSPPs modulates the microbial profile of the cecum while resulting in relatively less weight loss and lower rectal temperature compared to control.

## 1. Introduction

Helping to meet the ever-increasing demand for animal protein, chicken is categorized among one of the favorite protein sources for human consumption. Global warming has a bad impact on livestock production, which is indicated by the reduction in growth performance parameters in chickens [1,2]. It is important to minimize the after-effects of high ambient temperature in the livestock industry. Various strategies, such as thermal manipulation, in ovo feeding, improved managerial practices, nutrient modulation, etc., have been suggested to ameliorate heat stress (HS) effects [3,4,5,6]. Nutrient modulation and feed additives could be a reasonable solution for HS issues due to their ease of adaptation. Different supplements, such as vitamins, minerals, antioxidants, dietary fibers, prebiotics, probiotics, etc., have been used to modify the performance parameters of meat-type chicken [7,8,9]. Byproducts of various sources can also be used in livestock feeding to keep the costs low and to reduce the accumulation of waste. However, it is critical to evaluate the effects of such supplements when birds are exposed to high ambient temperatures.

Recent developments in the techniques employed to utilize byproducts from different sources may help make these inexpensive leftovers consumable and bring them to the consumable chain. The steam explosion method is one of these low-cost, environmentally friendly pretreatment methods that helps fractionate the biomass components by depolymerizing the lignin and hemicellulose into soluble monomers and oligomers [10,11]. Such products can then be utilized in various industries, including the livestock industry, as low-cost feed additives in the form of extracts [12,13]. This may have a double advantage: firstly, by reducing the cost of feed and, secondly, by providing fibrous and lignin contents in diets, which are required to modify the microbiota.

The cecum is the main site of anaerobic fermentation in which the breakdown of undigested fibrous material occurs in avian species [14]. Most plant-based bioproducts are rich in lignin and phenolic compounds, in addition to fiber. These substances are known for their effects on the proliferation of favorable bacteria and the abatement of pathogenic bacteria in the gut [15,16]. Lignin also has a prebiotic effect [15]. It has been suggested that the pathogenic microbial load in the gut of chickens may be increased under HS conditions [17]. However, since lignin and phenolic compounds can be obtained from inexpensive byproducts, such as solubles from shredded, steam-exploded pine particles (SSPPs), they may have some modulatory effects on the pathogenic microbes.

The present study was conducted to evaluate the effects of dietary SSPP supplementation on the performance and cecum microbiota in acute heat-stressed broiler chickens. We hypothesized that dietary supplementation of SSPP may fuel the beneficial microbiota of the chicken cecum, which might create heat tolerance in chickens.

## 2. Materials and Methods

The experiment was conducted at the animal research facility of the Gyeongsang National University, Korea. All the experimental procedures were approved by the Animal Ethics Committee of the Gyeongsang National University (GNU-200916-C0057).

### 2.1. Preparation of Solubles from Shredded, Steam-Exploded Pine Particles (SSPPs)

The preparation of SSPPs was undertaken by exploding pinewood chips of approximately 2 × 2 × 0.5 cm^3^ with steam at 200 °C for 11.5 min. The particles thus produced were used in our previous studies [17,18]. For use as a water extract (SSPP) in this study, these particles were mixed with water in a ratio of 46:100 (water to substance, *v*/*w*) and extracted at 80 °C for 213 min. The extract was then filtered through a Whatman filter paper, grade 2 (Z177601, Sigma-Aldrich Inc., Seoul, Korea), and stored at 4 °C until use. The SSPPs thus produced contained around 9.2% acid-insoluble lignin, 4.9% phenolic compounds, and 75.2% carbohydrates.

### 2.2. Experimental Birds and Housing

**Figure 1:**
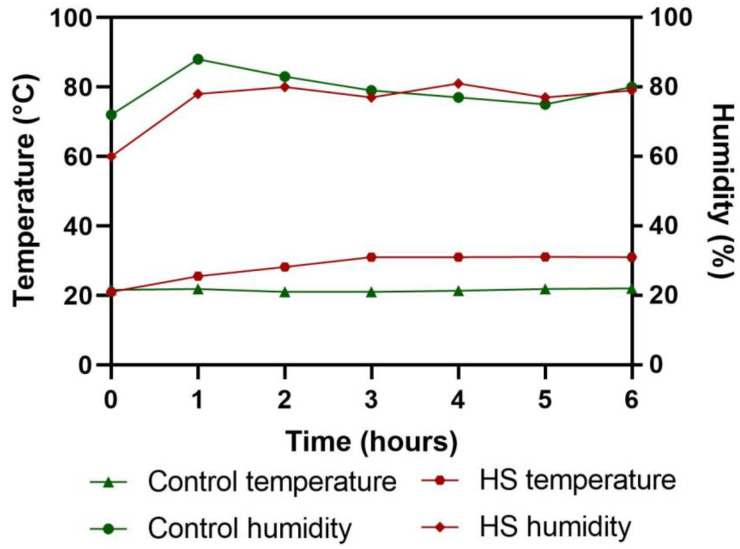
Changes in the temperature (°C) and relative humidity (%) of the rooms over the entire period of the experiment.

One-week-old Ross 308 broiler chicks (n = 108) with similar body weight (185.9 ± 1.2 g) were distributed into three dietary treatment groups of six replicates, with six birds in each replicate. The birds were reared in battery cages fitted with nipple-type waterers and trough-type feeders. The temperature of the room was maintained as per the recommendation of the Ross 308 broiler guide (Table S1). Exhaust fans were used for ventilation and room temperatures were controlled using automation. All birds were healthy and showed no signs of bacterial, viral, or parasitic infection. Commercial grower (8th day to 21st day) and finisher (22nd day to 37th day) feed containing 0, 0.1, and 0.4% solubles from shredded, steam-exploded pine particles (SSPPs) were fed to the birds. On the 37th day of age, a total of 40 birds (0% SSPP: 20 birds, 0.1% SSPP: 10 birds, 0.4% SSPP: 10 birds) near the average body weight (2490 ± 31 g) were selected and distributed into four groups of five replicates, with two birds in each replicate. One group (0% SSPP) was kept at thermoneutral temperature (NT; 21.0 °C) and served as a control, while the other three groups (0, 0.1, and 0.4% SSPPs) were heat-stressed in a separate room by gradually increasing the temperature of the room to 31 °C within the first three hours and then maintaining the same temperature for another three hours. The birds were exposed to HS once for six hours (Figure 1). The selection of the temperature and time for the acute HS was undertaken based on previous studies with slight modifications [17,19]. Finally, there were a total of four treatments: a control diet without any SSPP supplementation at thermoneutral temperature (0% NT), a control diet with acute heat stress (0% HS), a 0.1% SSPP-supplemented diet at AHS (0.1% HS), and a 0.4% SSPP-supplemented diet at AHS (0.4% HS). Feed and water were provided *ad libitum* throughout the experiment. Feed intake (FI), body weight (BW), and percent difference in body weight were calculated based on the data recorded before and after the HS experiment.

### 2.3. Rectal Temperature

Before and after the HS experiment, rectal temperature (RT) was recorded by inserting a digital thermometer (HI 91610, Hanna instruments Inc., Padova, Italy) up to approximately 3 cm inside the cloaca. The difference in the RT before and after HS is presented as ΔT.

### 2.4. Sampling and Measurements

A total of six birds from each treatment were randomly selected and euthanized using carbon dioxide after the HS experiment. Following a previously published procedure [20], blood samples were collected in a heparinized vacutainer through cardiac puncture and were centrifuged at 2000× *g* for 10 min at 4 °C to separate plasma. The plasma samples were then immediately stored at −20 °C until use.

The liver, spleen, and bursa of Fabricius were freely dissected and weighed, and the results are presented as relative to body weight (BW). Cecum samples were immediately collected in sterilized 50 mL falcon tubes and stored at −80 °C.

### 2.5. Plasma Analysis

The concentrations of plasma biochemical parameters, such as glucose, total protein, triglycerides, and total cholesterol, were analyzed using dry-slide technology in a VetTest Chemistry Analyzer (IDEXX Co., Ltd., Westbrook, ME, USA) following the manual instructions [21]. Briefly, the plasma samples were analyzed by installing the slides for glucose, total protein, triglyceride, and total cholesterol (IDEXX Co., Ltd., Westbrook, ME, USA), as well as plasma (70 µL) samples, in the analyzer. The results obtained from the automated analyzer were then recorded.

### 2.6. Cecum DNA Extraction and Metagenome Investigation

The extraction of cecal total genomic DNA was performed using a DNeasyPowerSoil Kit (Qiagen, Hilden, Germany) following the manufacturer’s instructions. The DNA samples were then quantified using Quant-IT PicoGreen (Invitrogen, Waltham, MA, USA). The 16S metagenomic sequencing library was constructed for metagenome assessment using a Herculase II Fusion DNA Polymerase Nextera XT Index Kit V2 (Illumina, San Diego, CA, USA). Illumina platform was used to sequence the library (Macrogen, Inc., Seoul, Korea). Quality profiling, adapter trimming, and read filtering for each sample were performed using the fastp program [22]. Sequences within the range of 400–500 bp were used and paired-end reads were assembled into one sequence using FLASH (v1.2.11) (Johns Hopkins University, Baltimore, USA) software [23]. The CD-HIT-EST program was used to determine the number of operational taxonomic units (OTUs) with a 97% sequence identity cutoff [24]. The BLAST+ (v2.9.0) (National Library of Medicine, Rockville Pike, Bethesda, MD, USA) program was then used to check taxonomic similarity against the reference database (NCBI 16S Microbial) [25]. Identical coverage of less than 85% was identified as not defined. QIIME (v1.9) (Northern Arizona University, Flagstaff, AZ, USA) software was used to evaluate the OTU abundance and obtain taxonomic information of the microbes. Alpha-diversity was assessed through the OTU, Chao1, Shannon, Good’s coverage, and inverse Simpson indices for the microbial species diversity and homogeneity. Beta diversity was assessed using unweighted/weighted Unifrac distances.

### 2.7. Statistical Analysis

Performance parameters related to growth were analyzed using ANCOVA. The parameters related to RT and organ weights and lengths were analyzed using a general linear model (GLM) procedure for the one-way ANOVA, followed by Duncan’s multiple range test. Planned contrasts were performed to compare 0% NT vs. 0% HS; 0% NT vs. 0.1% HS; 0% NT vs. 0.4% HS; 0% HS vs. 0.1% HS; 0% HS vs. 0.4% HS; 0% HS vs. 0.1% HS, and 0.4% HS; and 0% NT vs. 0% HS, 0.1% HS, and 0.4% HS. Microbial analyses, such as alpha-diversity and taxonomy (phylum and genus), were performed using the Kruskal–Wallis test and adjusted with Bonferroni correction. Differences were considered statistically significant at *p* < 0.05, except where otherwise stated. The beta diversity analysis is presented as a principal coordinate analysis (PCoA) based on unweighted and weighted Unifrac differences. PERMANOVA was used to evaluate the significant differences among treatments in the beta diversity. Spearman’s correlation was used to identify the correlations among bacterial phyla. All the data are expressed as means ± standard error of the mean (SEM). Analysis was performed using IBM SPSS Statistics package 25.0 (IBM software, Chicago, IL, USA) and SAS (SAS Institute Inc., Cary, NC, USA) software. Graphs were drawn using Prism-GraphPad (Graphpad Software, San Diego, CA, USA) software.

## 3. Results

The effects of different doses of SSPPs in diets on the growth performance parameters of birds kept at either NT or under acute heat stress are presented in Table 1. The ANCOVA results indicated that final BW and percent difference in BW were significantly affected, and HS birds had lower (*p* < 0.05) final BW and percent difference in BW irrespective of diets compared to birds kept at thermoneutral temperature. Final BW is positively related to the initial BW and had a significant effect (*p* < 0.001). The percent difference in BW has no relation to the initial BW. Contrast analysis revealed that the percent difference in BW was lower in HS birds in comparison to birds kept at thermoneutral temperature. The ANCOVA results indicated no variation in the feed intake. However, contrast analysis revealed that feed intake was decreased (*p* < 0.05) in HS birds in comparison to birds kept at thermoneutral temperature (0% NT vs. 0% HS, 0% NT vs. 0% HS, 0.1% HS, and 0.4% HS).

Compared with those birds kept at thermoneutral temperature, RT after six hours of HS and the difference in RT before and after HS (ΔT) were significantly increased (*p* < 0.001) in HS birds fed 0, 0.1, or 0.4% SSPPs in diets, with the highest values for 0.1% SSPP-supplemented chickens (Table 2). Contrast analysis revealed no significant variations (*p* > 0.05) among the treatment groups (0% HS vs. 0.1% HS and 0% HS vs. 0.4% HS) under HS conditions.

The relative organ weights of the liver, spleen, and bursa of Fabricius are presented in Table 3. No significant variations (*p* > 0.05) were observed in the liver, spleen, or bursa among the different treatment groups.

The plasma biochemical parameters, such as glucose, total protein, triglyceride, and cholesterol, are presented in Table 4. No significant variations (*p* > 0.05) were observed in the plasma biochemical parameters among the different treatment groups. Contrast analysis revealed that the level of triglyceride was decreased (*p* < 0.05) in HS birds (0% NT vs. 0% HS; 0% NT vs. 0.4% HS; 0% NT vs. 0% HS, 0.1% HS, and 0.4% HS) but was not affected (*p* > 0.05) in 0.1% HS in comparison to 0% NT birds.

**Figure 2:**
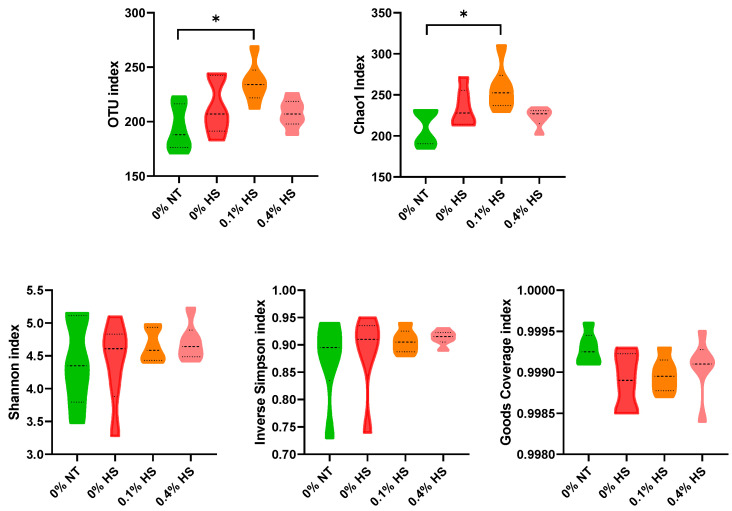
Effects of supplementing diets with solubles from shredded, steam-exploded pine particles (SSPPs) on the alpha-diversity (community richness and diversity) of cecum microflora in broiler chickens exposed to either thermoneutral or heat-stress conditions. Chickens were fed with diets containing 0% (control), 0.1%, and 0.4% SSPPs from the 8th day to the 36th day of age. On the 37th day, birds were either kept at thermoneutral temperature (21.0 °C) and provided a control diet (0% NT) or heat-stressed at 31.0 °C for six hours and provided a diet supplemented with 0% (0% HS), 0.1% (0.1% HS) or 0.4% (0.4% HS) SSPPs. Number of samples in treatment (n = 6). Abbreviations: OTUs, operational taxonomic unit; NT, normal temperature; HS, heat stress. *, significant difference at *p* < 0.05.

The alpha-diversity in the cecum samples was evaluated through community richness (OTUs and Chao1) and diversity (Shannon, inverse Simpson, and Good’s coverage indices) and is presented in Figure 2. The community richness evaluated through OTUs and Chao1 indices was significantly modified (*p* < 0.05) and higher in 0.1% HS in comparison to 0% NT birds. No significant variation (*p* > 0.05) was observed in the community diversity indices among the treatment groups.

**Figure 3:**
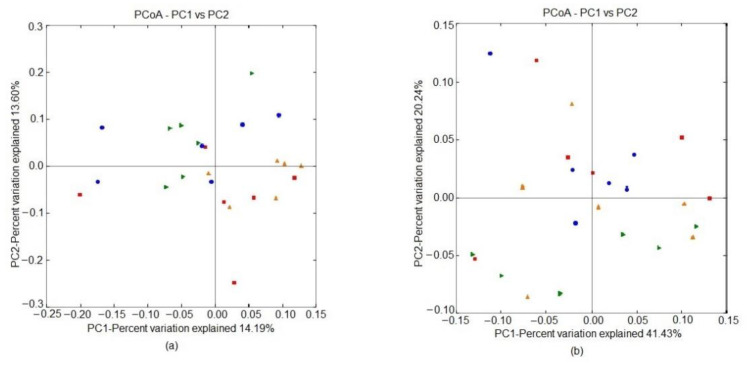
Effects of supplementing diets with solubles from shredded, steam-exploded pine particles (SSPPs) on the cecal microbiota composition assessed from unweighted (**a**) and weighted (**b**) Unifrac distances based on principal coordinate analysis (PCoA) in broiler chickens exposed to either thermoneutral or heat-stress conditions. Chickens were fed with diets containing 0% (control), 0.1%, and 0.4% solubles from shredded, steam-exploded pine particles (SSPPs) from the 8th day to the 36th day of age. On the 37th day, birds were either kept at thermoneutral temperature (21.0 °C) and provided a control diet (0% NT) or heat-stressed at 31.0 °C for six hours and provided a diet supplemented with 0% (0% HS), 0.1% (0.1% HS), or 0.4% (0.4% HS) SSPPs. Number of samples in each treatment (n = 6). Abbreviations: NT, normal temperature; HS, heat stress. The blue circle indicates 0% NT, the red square indicates 0% HS, the orange triangle indicates 0.1% HS, and the green triangle indicates 0.4% HS.

The PERMANOVA analysis of unweighted Unifrac distances showed significant variations among treatment groups (*p* < 0.05). The pairwise comparison revealed that the 0.1% HS group was different (*p* < 0.01) from the 0% NT and 0.4% HS groups (Figure 3a; Table 5). No variations (*p* > 0.05) were observed among the treatment groups in the weighted Unifrac distances (Figure 3b; Table 5).

**Figure 4:**
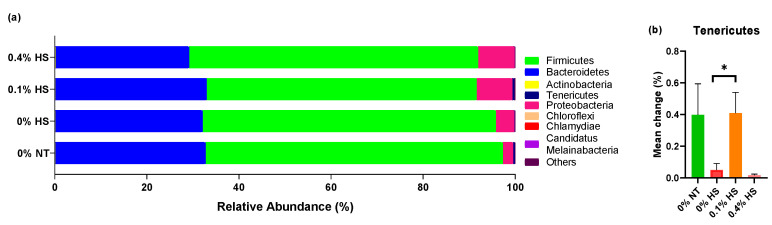
Effects of supplementing diets with solubles from shredded, steam-exploded pine particles (SSPPs) on the cecum microbial profiles of bacterial phyla (**a**) and significantly modified bacterial phyla (**b**) in broiler chickens exposed to either thermoneutral or heat-stress conditions. Chickens were fed with diets containing 0% (control), 0.1%, and 0.4% SSPP from the 8th day to the 36th day of age. On the 37th day, birds were either kept at thermoneutral temperature (21.0 °C) and provided a control diet (0% NT) or heat-stressed at 31.0 °C for six hours and provided a diet supplemented with 0% (0% HS), 0.1% (0.1% HS), or 0.4% (0.4% HS) SSPPs. Data show means ± SEM (n = 6). Abbreviations: NT, normal temperature; HS, heat stress. *, significant difference at *p* < 0.1.

To evaluate the effects of dietary SSPP and HS on the microbial community in the broiler cecum, the phyla and genera of the microbes were determined. *Firmicutes* and *Bacteroidetes* were the two most abundant phyla present in the chicken cecum (Figure 4a; Table S2). Kruskal–Wallis test indicated that *Tenericutes* was significantly (*p* < 0.05) modified among treatment groups and Bonferroni correction revealed its higher abundance (*p* = 0.08) in the 0.1% HS group in comparison to the 0% HS group (Figure 4b).

**Figure 5:**
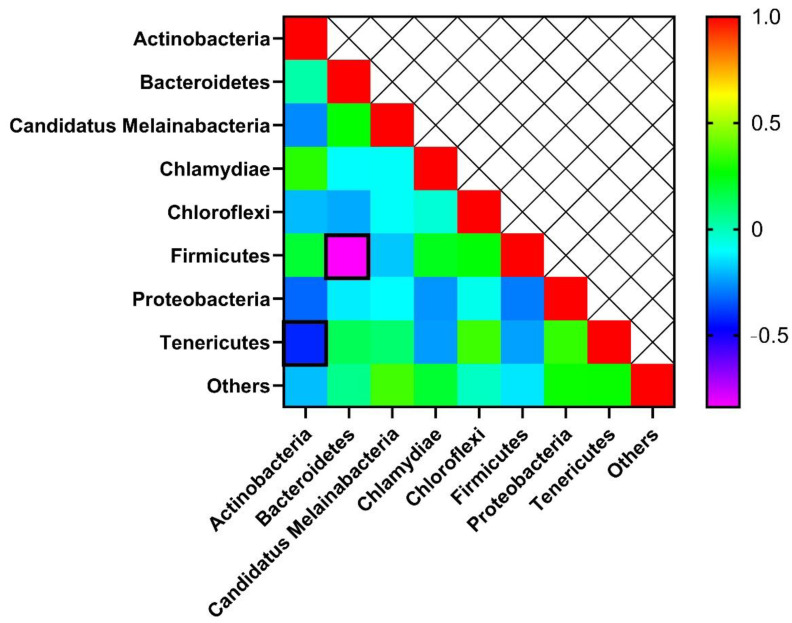
Cecum bacterial phyla correlation in broiler chickens. Zero represents similarity while plus and minus values represent positive and negative correlations, respectively. The square box outlined with black indicates significance at *p* < 0.05.

*Firmicutes* and *Bacteroidetes* were inversely correlated (Spearman R = −0.837, *p* = 0.001) with each other. Similarly, *Tenericutes* (Spearman R = −0.418, *p* = 0.042) was also inversely correlated with *Actinobacteria* (Figure 5).

**Figure 6:**
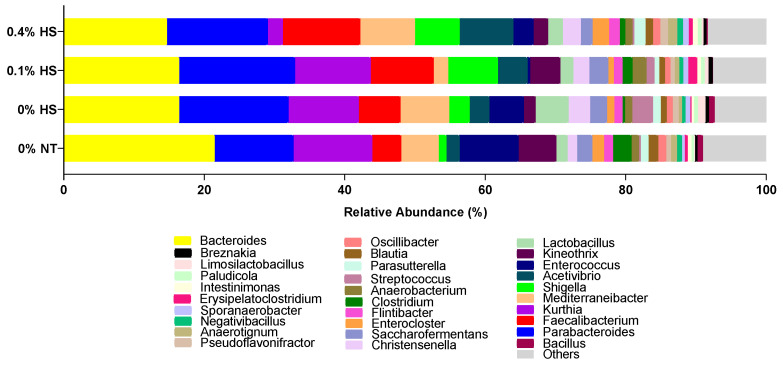
Effects of supplementing diets with solubles from shredded, steam-exploded pine particles (SSPPs) on the cecum microbial profiles of bacterial genera when broiler chickens were exposed to either thermoneutral or heat-stress conditions. Chickens were fed with diets containing 0% (control), 0.1%, and 0.4% SSPPs from the 8th day to the 36th day of age. On the 37th day, birds were either kept at thermoneutral temperature (21.0 °C) and provided a control diet (0% NT) or heat-stressed at 31.0 °C for six hours and provided a diet supplemented with 0% (0% HS), 0.1% (0.1% HS), or 0.4% (0.4% HS) SSPPs. Data show means (n = 6). Abbreviations: NT, normal temperature; HS, heat stress.

The cecum microbiota at the genus level is presented in Figure 6. *Bacteroides*, *Parabacteroides*, *Kurthia*, *Faecalibacterium, Mediterraneibacter*, and *Shigella* were the six most dominant genera in the chicken ceca. The abundance of *Bacteroides* was numerically lower while *Parabacteroides* and *Shigella* were increased in HS birds in comparison to birds kept at thermoneutral temperature. In terms of *Shigella*, only *S. sonnei* was detected in the chicken ceca on the 37th day of age.

**Figure 7:**
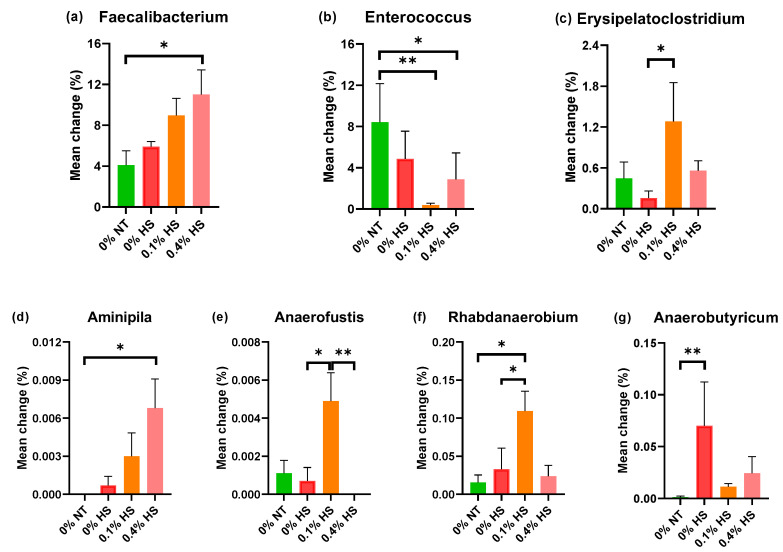
Effects of supplementing diets with solubles from shredded, steam-exploded pine particles (SSPPs) on the significantly modified genera *Faecalibacterium*, (**a**) *Enterococcus*, (**b**) *Erysipelatoclostridium*, (**c**) *Aminipila*, (**d**) *Anaerofustis*, (**e**) *Rhabdanaerobium*, (**f**) and *Anaerobutyricum* (**g**) in the ceca of broiler chickens exposed to either thermoneutral or heat-stress conditions. Chickens were fed with diets containing 0% (control), 0.1%, and 0.4% SSPPs from the 8th day to the 36th day of age. On the 37th day, birds were either kept at thermoneutral temperature (21.0 °C) and provided a control diet (0% NT) or heat-stressed at 31.0 °C for six hours and provided a diet supplemented with 0% (0% HS), 0.1% (0.1% HS), or 0.4% (0.4% HS) SSPPs. Data show means ± SEM (n = 6). Abbreviations: NT, normal temperature; HS, heat stress. * and **, significant differences at *p* < 0.1 and *p* < 0.05, respectively.

Among the top thirty genera, *Faecalibacterium*, *Enterococcus*, and *Erysipelatoclostridium* were significantly (*p* < 0.05) different among treatment groups (Figure 7). In terms of the genus *Enterococcus*, two species—namely, *E. cecorum* and *E. faecalis*—were detected in the cecum samples of 37 day old chicken. Furthermore, *Escherichia coli* was absent in the entire treatment group. The abundance of the genus *Faecalibacterium* was increased in 0.4% HS birds (*p* = 0.061), while the genus *Enterococcus*, including the species *E. cecorum* and *E. faecalis*, was decreased in 0.1% HS (*p* = 0.017) and 0.4% HS (*p* = 0.096) birds, respectively, in comparison to 0% NT birds. The other significantly modified (*p* < 0.05) genera present in the chicken ceca were *Aminipila*, *Anaerofustis*, *Rhabdanaerobium,* and *Anaerobutyricum*. *Aminipila*, and *Anaerobutyricum*, which were increased in the 0.4% HS (*p* = 0.052) and 0% HS (*p* = 0.016) groups in comparison to the 0% NT group. *Anaerofustis* was increased in the 0.1% HS group in comparison to the 0% HS (*p* = 0.079) and 0.4% HS (*p* = 0.012) groups, while *Rhabdanaerobium* was increased in the 0.1% HS group in comparison to the 0% NT (*p* = 0.071) and 0% HS (*p* = 0.09) groups, respectively.

## 4. Discussion

Alpha-diversity is an indicator of the richness and abundance of the microbial community expressed in OTUs and the Chao1 index. Increments in OTUs and the Chao1 index are correlated with better gut health in probiotic-fed chickens due to the proliferation of gut-favorable bacteria [26]. Unweighted Unifrac distances are used to represent the presence or absence of bacterial species. In the present study, in addition to significant modification of unweighted Unifrac distances in the 0.1% SSPP-supplemented diet (0.1% HS) compared to the 0% NT diet, the increase in OTUs and the Chao1 index might have been a result of the proliferation of advantageous bacteria, thus suggesting better intestinal strength. Previous studies also reported favorable bacteria-promoting activities for purified lignin [15]. The antipathogenic activity of phenolic compounds cannot be neglected as a beneficial effect of dietary SSPPs. They damage the cell membrane of the pathogenic bacteria and cause bacterial lysis [27]. Furthermore, in the present study, no significant variations were observed in the cecum community diversity indices, such as the Shannon, inverse Simpson, and Good’s coverage indices. In general, it is expected that HS tends to increase the penetration of pathogenic microbes in the intestine. The lack of variation in the cecum community diversity could have been due to the short period of stress, which lasted for six hours.

In the present study, *Firmicutes* and *Bacteroidetes* were the dominating phyla and were inversely correlated with each other, which is consistent with previous studies [18,28]. *Tenericutes* are among the bacterial phyla that have important roles in metabolism. Previous studies have suggested that the abundance of *Tenericutes* was reduced in unhealthy obese Chinese children [29]. Furthermore, *Tenericuute* abundance was found to be decreased in individuals with diminished insulin sensitivity [30]. In sum, the abundance of *Tenericutes* decreases under abnormal health conditions. The results of the present study revealed a numerically lower abundance of *Tenericutes* in heat-stressed chickens (0% HS vs. 0% NT). Furthermore, the abundance of *Tenericutes* was significantly increased in 0.1% HS in comparison to 0% HS birds, demonstrating better health. This indicates that supplementation with 0.1% SSPPs helps the proliferation of *Tenericutes* even under HS conditions. The reason behind this could be related to the availability of a higher amount of hexoses in SSPPs, which was also demonstrated through the numerically higher plasma glucose levels in 0.1% SSPP-supplemented chickens. As chickens have a higher insulin tolerance, the proliferation of *Tenericutes* in the cecum of HS chickens may have been facilitated [31]. In contrast, *Actinobacteria* are negatively correlated with the serum glucose levels in porcine [32]. In conclusion, *Tenericutes,* and *Actinobacteria* should be inversely correlated with each other, which confirms our present finding in chickens.

In the present study, *Bacteroides* and *Parabacteroides* were the two most dominant genera and were numerically decreased and increased, respectively, in HS birds. This correlates with previous studies, which have shown a similar pattern in their abundances under HS [33,34]. *Shigella* is another genus that was increased in HS birds. The bacterium *S. sonnei* was the only species present in the cecum samples. The bacterium *S. sonnei* is associated with infection and disease in chickens due to its colonization of the intestinal epithelial cells [35]. A numerically higher abundance of *S. sonnei* in the HS birds indicates that even short-term heat exposure can trigger pathogenic bacterial infiltration.

*Faecalibacterium* is known to produce butyrate, which inhibits pathogenic microbes and is thus linked with health benefits in poultry [36,37]. In the present study, the abundance of *Faecalibacterium* was significantly increased in 0.4% HS birds in comparison to 0% NT birds. The exact reason behind the increase in the abundance of *Faecalibacterium* in 0.4% HS birds is not clear. However, it can be speculated that the supplementation with SSPPs may have helped the proliferation of *Faecalibacterium* in the cecum. The abundance of *Faecalibacterium* is positively correlated with butyrate production [38] and helps strengthen the mucosal layer by modifying the goblet cells and mucin in the intestine [39]. It is already established that HS tends to negatively impact gut health by damaging the mucosal layer [40]. An increase in the butyrate-producing *Faecalibacterium* genus may consolidate the mucosal layer in the intestine, indicating better gut health in HS birds supplemented with 0.4% SSPPs in their diets.

The genus *Enterococcus* comprises lactic acid-producing commensal bacteria that belong to the phylum *Firmicutes* and are partially harmful due to their low pathogenic tendencies [41,42]. In the present study, *E. cecorum* and *E. faecalis* were the only two species identified in the cecum samples that were significantly decreased in SSPP-supplemented groups (0% NT vs. 0.1% HS and 0% NT vs. 0.4% HS) in comparison to thermoneutral control. The presence of *E. cecorum* and *E. faecalis* is common in poultry but its increased prevalence is associated with infection and disease [43]. The abundance of *Enterococcus* was found to increase in a temperature-dependent manner, with the highest abundance in high-temperature conditions [34]. In the present study, we did not find significant variation in the abundances of *Enterococcus* between the chickens kept at thermoneutral (0% NT) and HS (0% HS) conditions. This could have been due to the exposure of the chickens to HS at lower intensity for a shorter period of time. However, a decrease in the *Enterococcus* in SSPP-supplemented diets indicated the beneficial effect of SSPP supplementation in chickens.

In the present study, the genus *Anaerofustis* was increased in 0.1% SSPP (0.1% HS) birds in comparison to 0% HS and 0.4% HS birds. *Anaerofustis* is known to be positively correlated with the presence of carbohydrates (xylitol) in mice [44]. The SSPPs used in the present study are also rich in carbohydrate content, due to which a higher abundance of *Anaerofustis* was noticed in the ceca of 0.1% HS chickens.

The genus *Anaerobutyricum* is a fibrinolytic bacterium that has been reclassified as *Eubacterium* [45]. Although we did not find any significant variation in the abundance of *Anaerobutyricum* in SSPP-supplemented diets, its abundance was significantly increased in the HS birds (0% HS) in comparison to the control. This is in correlation with previous studies, where the abundance of *Eubacterium* increased in heat-stressed rabbits and gestation sows [46,47].

*Erysipelatoclostridium*, *Aminipila,* and *Rhabdanaerobium* were the three other significantly modified genera in this study. The role of *Erysipelatoclostridium* is associated with susceptibility to infection in humans [48]. However, information about *Erysipelatoclostridium* is limited in chickens. The cases of *Aminipila* and *Rhabdanaerobium* are similar, as they are novel bacterial genera that require further exploration.

Broilers are reared as a meat source for the production of animal protein. Precise nutritional practices have enabled broilers to attain maximum weight in the minimum time. However, HS has a detrimental effect on chickens [1,2,20]. Countries that import most of their animal feed constituents need to reduce their dependence on other countries by utilizing less commonly used domestic feed sources, such as industrial byproducts. A recent study suggested that wood industry-related byproducts can be utilized in chicken feed as a source of fiber without any adverse effects [18]. Therefore, the present study was conducted to evaluate the effects of dietary SSPPs on the performance and metagenome of broilers exposed to short-term acute HS. The final BW and the percent difference in BW were significantly decreased in birds exposed to HS in comparison to birds kept at NT. This corroborates previous studies, where HS tended to decrease BW gain and percent difference in BW when birds were exposed to 31 °C for ten hours and six hours, respectively [17,49]. Under high-temperature conditions, it has been suggested that birds may face problems in releasing body heat due to a lack of sweat glands and, thus, demonstrate panting behavior [20]. This may keep the bird away from feed, resulting in decreasing feed intake under HS [2,17]. Interestingly, in the present study, contrast analysis revealed a significant decrease in feed intake in the 0% HS treatment, and it was not affected in SSPP treatments (0.1% HS and 0.4% HS), in comparison to the 0% NT treatment. This suggests that birds supplemented with 0.1% and 0.4% SSPPs in diet were undergoing comparatively less HS than those without supplementation with SSPPs, indicating a higher heat tolerance capacity in SSPP-fed chickens.

Rectal temperatures increase under HS and can be used as a marker under high ambient temperature conditions [17,50]. The present study also indicated an increase in RT under HS. Heat exchange with the surroundings is critical for modulating RT [51]. Increases in RT could be attributable to the lower rate of heat exchange with the surroundings and higher respiration rates under HS, partly due to the lack of sweat glands in chickens [52]. The relatively small increase in RT and ΔT in 0.4% HS birds compared to 0.1% HS birds suggested that birds supplemented with 0.4% SSPPs are able to better tolerate HS in this experimental setting. Although we did not evaluate the respiration rates in this study, the possibility of lower respiration rates with 0.4% SSPPs exists and could be explored in the future.

The liver plays an important role in metabolism and is susceptible to external stressors, leading to hemostasis disruption [53]. However, there are contradictory results in terms of the relative body weight of the liver under HS. For instance, a study conducted on broilers suggested an increase in the relative liver weight on the 7th and 14th days post-HS when birds were exposed to 32 °C from 28 to 42 days of age [54]. On the other hand, the relative liver weight decreased in birds exposed to 34 °C from 22 to 42 days of age [55]. In the present study, we did not find any significant effects on the relative liver weight on the 37th day of age when birds were exposed to 6 h of HS. There are various potential reasons behind this, including the age of the birds and the intensity of HS and exposure time. Lymphoid organs are responsible for imparting immunity in chickens. Long-term HS tends to decrease lymphoid organ (bursa of Fabricius and spleen) weights in chickens [1]. Generally, the atrophy of lymphoid organs is connected with a succession of illnesses, such as undernourishment, inflammation, and oxidative stress [56]. The lack of variation in the relative weight of the bursa of Fabricius and spleen in this study might have been due to the short time of acute HS.

Glucose and cholesterol are responsible for carbohydrate and lipid metabolism in chickens. The level of cholesterol increases while that of total protein remains unchanged under HS [57]. The level of glucose is quite unstable under HS, either being increased [57,58] or remaining unchanged [19]. An increase in the concentration of glucose can be looked upon as a surviving factor under HS [57]. This indicates that an increment in the level of glucose might be related to the exposure time. In the present study, no significant variations were reported in the plasma glucose, total protein, or cholesterol levels in HS birds, which could have been due to a short exposure (six hours) to acute HS, which may not have been sufficient enough to induce glucose in chickens. Triglycerides are lipids that are stored in the body for energy production. In the present study, contrast analysis revealed a decrease in the serum triglyceride levels in heat-exposed chickens in comparison to thermoneutral control. This is in correlation with previous studies where HS was found to decrease serum triglyceride in ducks [59]. However, the serum triglyceride levels in 0.1% SSPP-supplemented birds (0.1% HS) were similar to those of 0% NT birds and were not affected by HS. This could be seen as a positive effect of supplementing diets with SSPPs and might have been due to the presence of a high concentration of carbohydrates in the SSPPs. As discussed above, a decrease in the plasma triglyceride levels might be connected to the higher fat metabolism needed to meet the excessive energy demand under HS conditions. The panting behavior makes the feed inaccessible to the birds, which was confirmed by the reduction in feed intake in HS birds. The lack of significant variations in 0% NT vs. 0.1% HS birds in the feed intake indicates that dietary SSPP-supplemented birds consumed similar feed. Furthermore, higher carbohydrate levels in the SSPPs (0.1% HS) may have been substituted for the energy requirement, leading to reduction in triglyceride hydrolysis.

## 5. Conclusions

The community richness indicators, such as OTUs and Chao1, were increased in 0.1% HS birds and accompanied by modified unweighted Unifrac distances, indicating that enhanced production of favorable bacteria might have been due to the presence of lignin and phenolic compounds in SSPPs. Chickens supplemented with SSPPs showed increased abundance of the phylum *Tenericutes* and the genera *Faecalibacterium* and *Anaerofustis*, which are favorable for the gut, but decreased abundance in the partially virulent genus *Enterococcus*. Thus, it can be suggested that dietary supplementation of SSPPs can improve gut microbiota and alleviate body temperature in broilers exposed to acute HS.

## Figures and Tables

**Table 1:** Effects of supplementing diets with solubles from shredded, steam-exploded pine particles (SSPPs) on the growth performances in broiler chickens exposed to either thermoneutral or heat-stress conditions.

		Final BW (g)	% Difference in BW	Feed Intake (g/Bird)
Treatments	0% NT	2535 b ± 13	1.79 b ± 0.53	62 ± 5.6
	0% HS	2454 a ± 13	−1.46 a ± 0.53	44 ± 5.6
	0.1% HS	2466 a ± 13	−0.95 a ± 0.53	46 ± 5.6
	0.4% HS	2476 a ± 13	−0.56 a ± 0.53	47 ± 5.6
	*p* value	0.003	0.003	0.128
Initial BW	Est	0.956	−0.002	0.025
	SE	0.109	0.004	0.045
	*p* value	0.001	0.679	0.586
Contrast *p* values	0% NT vs. 0% HS	0.068	0.001	0.028
	0% NT vs. 0.1% HS	0.305	0.002	0.070
	0% NT vs. 0.4% HS	0.181	0.005	0.066
	0% HS vs. 0.1% HS	0.385	0.541	0.647
	0% HS vs. 0.4% HS	0.586	0.238	0.665
	0% HS vs. 0.1% HS, and 0.4% HS	0.415	0.301	0.607
	0% NT vs. 0% HS, 0.1% HS, and 0.4% HS	0.091	0.001	0.020

Chickens were fed diets containing 0% (control), 0.1%, or 0.4% solubles from shredded, steam-exploded pine particles (SSPPs) from the 8th day to the 36th day of age. On the 37th day, birds were either kept at thermoneutral temperature (21.0 °C) and provided a control diet (0% NT) or heat-stressed at 31.0 °C for six hours and provided a diet supplemented with 0% (0% HS), 0.1% (0.1% HS), or 0.4% (0.4% HS) SSPPs. Data show least-square means ± SEM (n = 5). a, b: Different letters indicate significant differences (*p* < 0.05). Abbreviations: BW, body weight; NT, normal temperature; HS, heat stress; Est, estimate; SE, standard error.

**Table 2:** Effects of supplementing diets with solubles from shredded, steam-exploded pine particles (SSPPs) on the rectal temperature in broiler chickens exposed to either thermoneutral or heat-stress conditions.

Treatments	Rectal Temperature (°C)		
	Before	After	ΔT
0% NT	40.9 ± 0.15	41.3 a ± 0.10	0.4 a ± 0.15
0% HS	41.2 ± 0.18	43.6 bc ± 0.23	2.4 bc ± 0.29
0.1% HS	41.1 ± 0.13	44.0 c ± 0.45	2.9 c ± 0.36
0.4% HS	41.1 ± 0.10	42.9 b ± 0.13	1.7 b ± 0.17
*p* value	0.565	0.001	0.001
Contrast *p* values			
0% NT vs. 0% HS	0.178	0.001	0.001
0% NT vs. 0.1% HS	0.433	0.001	0.001
0% NT vs. 0.4% HS	0.330	0.001	0.002
0% HS vs. 0.1% HS	0.555	0.302	0.173
0% HS vs. 0.4% HS	0.693	0.066	0.089
0% HS vs. 0.1% HS and 0.4% HS	0.570	0.608	0.827
0% NT vs. 0% HS, 0.1% HS, and 0.4% HS	0.208	0.001	0.001

Chickens were fed with diets containing 0% (control), 0.1%, or 0.4% solubles from shredded, steam-exploded pine particles (SSPPs) from the 8th day to the 36th day of age. On the 37th day, birds were either kept at thermoneutral temperature (21.0 °C) and provided a control diet (0% NT) or heat-stressed at 31.0 °C for six hours and provided a diet supplemented with 0% (0% HS), 0.1% (0.1% HS), or 0.4% (0.4% HS) SSPP. “Before” and “After” indicate rectal temperatures taken before starting and after undergoing six hours of heat stress, respectively. ΔT indicates the difference in temperature before and after heat stress. Data show means ± SEM (n = 5). a–c: Different letters indicate significant differences (*p* < 0.05). Abbreviations: NT, normal temperature; HS, heat stress.

**Table 3:** Effects of supplementing diets with solubles from shredded, steam-exploded pine particles (SSPPs) on the relative organ weight (% body weight) in broiler chickens exposed to either thermoneutral or heat-stress conditions.

	Relative Organ Weight (%)		
Treatment	Liver	Spleen	Bursa
0% NT	2.83 ± 0.06	0.12 ± 0.01	0.10 ± 0.02
0% HS	2.60 ± 0.17	0.14 ± 0.01	0.09 ± 0.01
0.1% HS	2.60 ± 0.16	0.15 ± 0.01	0.10 ± 0.01
0.4% HS	2.51 ± 0.19	0.13 ± 0.01	0.12 ± 0.01
*p* value	0.493	0.436	0.522
Contrast *p* values			
0% NT vs. 0% HS	0.291	0.247	0.595
0% NT vs. 0.1% HS	0.294	0.139	0.905
0% NT vs. 0.4% HS	0.147	0.610	0.346
0% HS vs. 0.1% HS	0.995	0.732	0.517
0% HS vs. 0.4% HS	0.676	0.507	0.148
0% HS vs. 0.1% HS and 0.4% HS	0.812	0.852	0.226
0% NT vs. 0% HS, 0.1% HS, and 0.4% HS	0.149	0.199	0.826

Chickens were fed with diets containing 0% (control), 0.1%, and 0.4% solubles from shredded, steam-exploded pine particles (SSPPs) from the 8th day to the 36th day of age. On the 37th day, birds were either kept at thermoneutral temperature (21.0 °C) and provided a control diet (0% NT) or heat-stressed at 31.0 °C for six hours and provided a diet supplemented with 0% (0% HS), 0.1% (0.1% HS), or 0.4% (0.4% HS) SSPPs. Data show means ± SEM (n = 6). Abbreviations: NT, normal temperature; HS, heat stress.

**Table 4:** Effects of supplementing diets with solubles from shredded, steam-exploded pine particles (SSPPs) on the blood metabolites in broiler chickens exposed to either thermoneutral or heat-stress conditions.

Blood Biochemicals (mg/dl)				
Treatment	Glucose	Total Protein	Triglyceride	Cholesterol
0% NT	295.5 ± 24.9	3.3 ± 0.18	47.5 ± 11.5	139.3 ± 8.5
0% HS	326 ± 16.4	3.5 ± 0.17	24 ± 3.6	135.5 ± 8.0
0.1% HS	370.3 ± 45.8	3.8 ± 0.27	31 ± 6.2	129.3 ± 7.9
0.4% HS	305.3 ± 29.7	3.9 ± 0.41	25.2 ± 5.4	152 ± 22.4
*p* value	0.357	0.408	0.121	0.672
Contrast *p* values				
0% NT vs. 0% HS	0.496	0.704	0.034	0.840
0% NT vs. 0.1% HS	0.104	0.279	0.127	0.599
0% NT vs. 0.4% HS	0.825	0.129	0.043	0.507
0% HS vs. 0.1% HS	0.325	0.476	0.507	0.745
0% HS vs. 0.4% HS	0.643	0.245	0.911	0.389
0% HS vs. 0.1% HS and 0.4% HS	0.759	0.280	0.654	0.753
0% NT vs. 0% HS, 0.1% HS, and 0.4% HS	0.298	0.223	0.023	0.980

Chickens were fed with diets containing 0% (control), 0.1%, and 0.4% solubles from shredded, steam-exploded pine particles (SSPPs) from the 8th day to the 36th day of age. On the 37th day, birds were either kept at thermoneutral temperature (21.0 °C) and provided a control diet (0% NT) or heat-stressed at 31.0 °C for six hours and provided a diet supplemented with 0% (0% HS), 0.1% (0.1% HS), or 0.4% (0.4% HS) SSPPs. Data show means ± SEM (n = 6). Abbreviations: NT, normal temperature; HS, heat stress.

**Table 5:** Effects of supplementing diets with solubles from shredded, steam-exploded pine particles (SSPPs) on the beta diversity (PERMANOVA) as indicated by unweighted and weighted Unifrac distances in broiler chickens exposed to either thermoneutral or heat-stress conditions.

	PERMANOVA	
	Unweighted Unifrac Distances	Weighted Unifrac Distances
Test statistic	1.352	0.926
*p* value	0.02	0.469
Number of permutations	999	999
Pairwise comparison		
0% NT vs. 0% HS	0.183	0.983
0% NT vs. 0.1% HS	0.007	0.397
0% NT vs. 0.4% HS	0.610	0.122
0% HS vs. 0.1% HS	0.295	0.576
0% HS vs. 0.4% HS	0.063	0.273

Chickens were fed with diets containing 0% (control), 0.1%, and 0.4% solubles from shredded, steam-exploded pine particles (SSPPs) from the 8th day to the 36th day of age. On the 37th day, birds were either kept at thermoneutral temperature (21.0 °C) and provided a control diet (0% NT) or heat-stressed at 31.0 °C for six hours and provided a diet supplemented with 0% (0% HS), 0.1% (0.1% HS), or 0.4% (0.4% HS) SSPPs. Number of samples in each treatment (n = 6). Abbreviations: NT, normal temperature; HS, heat stress.

## Data Availability

The data presented in this study are available on request from the corresponding author.

## Supplementary Materials

The following supporting information can be downloaded at: https://www.mdpi.com/article/10.3390/microorganisms10091795/s1, Table S1: The temperature of the room was maintained from the first week of age until the start of the acute heat stress experiment. The relative humidity was maintained at 50% to 60% throughout the experiment. Table S2: Effects of supplementing diets with solubles from shredded, steam-exploded pine particles (SSPP) on the most prevalent Bacteroidetes and Firmicutes in the cecum of broiler chickens exposed to either thermoneutral or heat-stress conditions.
