# Supplementary material for: Effects of Dietary Supplementation of Solubles from Shredded, Steam-Exploded Pine Particles on the Performance and Cecum Microbiota of Acute Heat-Stressed Broilers
Source: Microorganisms. 2022 Sep 6;10(9):1795. doi: 10.3390/microorganisms10091795 (PMC9504121; doi:10.3390/microorganisms10091795)
Supplement: Supplementary file 1 [file microorganisms-10-01795-s001.zip › microorganisms-1881454-supplementary.pdf]

Table S1. The temperature of the room was maintained from the first week of age until the start of the acute heat stress experiment. The relative humidity was maintained at 50% to 60% throughout the experiment.

Age (day)	Temperature °C
7	27.7 – 29.9
9	26.7 – 28.6
12	25.7 – 27.8
15	24.8 – 26.8
18	23.6 – 25.5
21	22.7 – 24.7
24	21.7 – 23.5
27 and thereafter	20.7 – 22.7

Table S2. Effects of supplementing diets with solubles from shredded, steam-exploded pine particles (SSPP) on the most prevalent Bacteroidetes and Firmicutes in the cecum of broiler chickens exposed to either thermoneutral or heat-stress conditions.

**Phylum**	**Genus**	**0% NT**	**0% HS**	**0.1% HS**	**0.4% HS**
Bacteroidetes	Bacteroides	21.4	16.4	16.4	14.7
	Parabacteroides	11.2	15.6	16.5	14.4
Firmicutes	Kurthia	11.2	10.0	10.8	2.1
	Faecalibacterium	4.1	5.9	9.0	11.0
	Mediterraneibacter	5.3	7.0	2.1	7.8
	Acetivibrio	1.9	2.8	4.2	7.7
	Enterococcus	8.4	4.9	0.4	2.9
	Kineothrix	5.3	1.7	4.3	2.0
	Lactobacillus	1.6	4.7	1.8	2.1
	Christensenella	1.3	3.0	2.3	2.6
	Saccharofermentans	2.2	2.4	2.7	1.7
	Enterocloster	1.6	1.0	0.8	2.3

Chickens were fed with diets containing 0% (control), 0.1%, and 0.4% solubles from shredded, steam-exploded pine particles (SSPP) from the 8th day to the 36th day of age. On 37th day, birds were either kept at thermoneutral temperature (21.0 °C) and provided control diet (0% NT) or heat-stressed at 31.0 °C for six hours and supplemented with 0% (0% HS), 0.1% (0.1% HS) and 0.4% (0.4% HS) SSPP in diets. The table represents the top two Bacteroidetes and the top ten Firmicutes. Data show mean ± SEM (n = 6). Abbreviations: NT, normal temperature; HS, heat stress.
